# Proton therapy in Asia Pacific: current resources, international disparities and steps forward

**DOI:** 10.1002/jmrs.776

**Published:** 2024-02-29

**Authors:** Luisa E. Jacomina, Ryan Anthony F. Agas, Edward Christopher Dee, Pankaj Kumar Panda, Michael Benedict A. Mejia

**Affiliations:** ^1^ Division of Radiation Oncology The University of Texas MD Anderson Cancer Centre Houston Texas USA; ^2^ Department of Radiation Oncology, Benavides Cancer Institute University of Santo Tomas Hospital Manila Philippines; ^3^ Department of Radiation Oncology Memorial Sloan Kettering Cancer Centre New York New York USA; ^4^ Clinical Research Secretariat, Apollo Proton Cancer Centre Chennai India

## Abstract

The burden of cancer in Asia Pacific, a region home to over four billion people, is growing. Because of sheer demographics alone, the Asia Pacific region arguably has the highest number of patients who can benefit from protons over conventional x‐rays. However, only 39 out of 113 proton facilities globally are in Asia Pacific, and 11 of them are in low‐ and middle‐income countries where 95% of the regional population reside. We draw attention to present resource distribution of proton therapy in Asia Pacific, highlight disparities in access, and suggest steps forward.

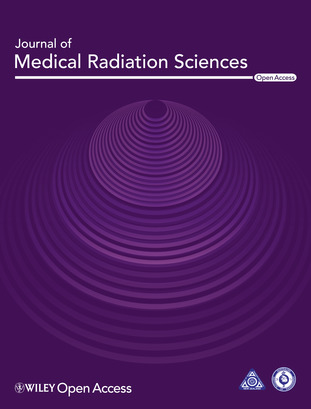

The burden of cancer in Asia Pacific, a region home to over 4 billion people, is growing. More than half of new cancer cases and cancer deaths in the world occur in the region annually, most commonly lung, breast and colorectal cancer. Of interest, the Asia Pacific accounts for ~75% of liver, head and neck (especially nasopharynx), and oesophageal cancers globally – sites that are active areas of investigation for proton therapy.^1^ Here, we draw attention to the present resource distribution of particle therapy, particularly proton therapy, in the region, highlight disparities in access and suggest steps forward.

The Asia Pacific region includes more than half of the world's population. Although there are various definitions as to which countries constitute Asia Pacific, it generally includes countries in mainland Asia and archipelagos surrounding the Western Pacific Ocean. The United Nations' definition, through the Economic and Social Commission for Asia and the Pacific (ESCAP), identifies 58 constituent countries in five distinct subregions.^2^ The Asia Pacific is characterised by immense sociocultural diversity and rapid economic growth, with marked heterogeneity in history, religion, language, politics and health systems.

Proton beam therapy (PBT) uses a beam of positively charged particles, or protons, to deliver therapeutic radiation. Compared with conventional x‐rays, protons deposit the radiation dose to a specified depth in the body, reducing dose to adjacent organs by as much as 50–70%.^3^ Because of its unique physical properties, protons are associated with a lower risk of adverse events and improved patient outcomes in various clinical settings. The evidence supporting PBT continues to grow, although its clinical use differs geographically depending on country‐specific regulations and policy‐approved indications.

Despite growing awareness of the potential clinical advantages of PBT over conventional x‐rays, the global PBT utilisation remains highly variable. On one hand, there are centres operating at full or even over capacity with significant waiting times. On the other hand, there are centres that are either shutting down or treating more common malignancies without definite evidence for PBT in order to maintain revenue. While multifactorial and beyond the scope of this article, the issues surrounding PBT's struggle to gain widespread traction are centred around the added cost and complexity relative to the incremental clinical benefit, rather than the absolute number of PBT‐eligible patients. Worldwide estimates in 2020 revealed that the number of patients who were treated with PBT was only less than 2% of those who were likely to benefit from it.^4^

There is an extreme imbalance in the distribution of proton facilities that impedes global access to PBT. Establishing proton facilities is associated with staggering costs and a high physical space requirement due to the size and complexity of PBT equipment. While a conventional x‐ray system can cost ~US$6 million in capital expenditure, a single‐room proton treatment unit can cost up to US$30–50 million.^4^ This drives up treatment costs to as much as three to four times that of a similar course using x‐rays, raising concerns on its profitability and cost‐effectiveness.^5^ With the paucity of high‐level evidence showing a clear benefit for protons in many common clinical settings, limited insurance coverage and high out‐of‐pocket expenses further discourage eligible patients from receiving PBT. These factors make it difficult to justify the hefty investment in establishing a proton facility especially in developing countries already struggling with basic cancer treatment facilities as is. It comes as no surprise that 102 out of the 113 proton facilities in the world are in high‐income countries (gross national income [GNI] per capita >US$13,846).^6^, ^7^

There are 39 proton facilities in operation in Asia Pacific as of October 2023 (Table 1). They are distributed in nine countries, namely China, Hong Kong, India, Japan, Russia, Singapore, South Korea, Thailand and Taiwan. Of note, China, Russia, and Thailand are upper middle‐income countries (GNI per capita US$4466 to US$13,846), and India is a lower middle‐income country (GNI per capita US$1136 to US$4465).^7^, ^8^ In effect, there are only 11 facilities in Asia Pacific, and the world, not located in a high‐income country. The large discrepancy in the availability of proton facilities between high‐ and low‐ to middle‐income countries (LMIC) magnifies the inequity in access to high‐quality comprehensive cancer care in LMICs. This is important to address because 95% of the Asia Pacific population reside in LMICs; and they experience a disproportionate burden of cancer, projected to account for nearly 75% of global cancer deaths by 2030.^9^, ^10^

**Table 1:** Proton radiotherapy facilities in operation in the Asia Pacific region as of October 2023.^7^

Country	Proton facility	Start of operations
China	WPTC, Wanjie, Zi‐Bo	2004
China	SPHIC, Shanghai	2014
China	Ruijin Hospital, Jiao Tong University, Jiading, Shanghai	2021
China	Hefei Ion Medical Center, Hefei, Anhui	2022
Hong Kong	Hong Kong Sanatorium and Hospital PTC, Hong Kong	2023
India	Apollo Proton Cancer Centre, Chennai	2019
Japan	NCC, Kashiwa	1998
Japan	HIBMC, Hyogo	2001
Japan	PMRC 2, Tsukuba	2001
Japan	Shizuoka Cancer Center	2003
Japan	STPTC, Koriyama‐City	2008
Japan	MPTRC, Ibusuki	2011
Japan	Fukui Prefectural Hospital PTC, Fukui City	2011
Japan	Nagoya PTC, Nagoya City, Aichi	2013
Japan	Hokkaido Univ. Hospital PBTC, Hokkaido	2014
Japan	Aizawa Hospital PTC, Nagano	2014
Japan	Tsuyama Chuo Hospital, Okayama	2016
Japan	PTC Teishinkai Hospital, Sapporo, Hokkaido	2016
Japan	Hakuhokai Group Osaka PT Clinic, Osaka	2017
Japan	Kobe Proton Center, Kobe	2017
Japan	Narita Memorial Proton Center, Toyohgashi	2018
Japan	Hokkaido Ohno Memorial Hospital, Sapporo	2018
Japan	Takai Hospital, Tenri City	2018
Japan	Nagamori Memorial Center of Innovative Cancer Therapy and Research, Kyoto	2019
Japan	Shonan Kamakura Advanced Medical Center, Kanagawa	2022
Russia	ITEP, Moscow	1969
Russia	JINR 2, Dubna	1999
Russia	MIBS, Saint‐Petersburg	2018
Russia	MRRC, Obninsk	2016
Russia	Federal HighTech Center of FMBA, Dimitrovgrad	2019
Singapore	Mount Elizabeth Novena Hospital Proton Center	2023
Singapore	National Cancer Center Singapore (NCCS)	2023
Singapore	Singapore Institute for Advanced Medicine	2023
South Korea	KNCC, IIsan	2007
South Korea	Samsung PTC, Seoul	2015
Thailand	Her Royal Highness Princess Chakri Sirindhorn PC, Bangkok	2021
Taiwan	Chang Gung Memorial Hospital, Taipei	2015
Taiwan	Chang Gung Memorial Hospital, Kaohsiung	2018
Taiwan	Taipei Medical University Hospital (TMUH), Taipei	2022

Although widespread availability and equitable access to PBT in Asia Pacific remains elusive, steps are being taken globally to improve affordability of proton systems.^4^ Efforts are aimed at developing compact systems that fit the standard treatment room of a linear accelerator which will lower capital costs and enable more affordable treatments. As PBT gains worldwide traction, clinical trials determining appropriate indications for protons are also anticipated to increase in parallel. There are 19 proton facilities in Asia Pacific expected to be completed over the coming years; these present opportunities to encourage clinical trial participation within the region and in collaboration with the rest of the world.^11^ PBT trials conducted in Asia Pacific are necessary to generate evidence applicable to the unique yet diverse patient population in the region and contribute to the overall learning curve for PBT.

Because of sheer demographics alone, the Asia Pacific region arguably has the highest number of patients who can benefit from protons over conventional x‐rays. The magnitude of need for PBT has been estimated for many European countries, ranging from 10% to 16% of all patients receiving radiotherapy.^12^ No such estimate exists for the Asia Pacific region; but conservative and generous estimates for LMICs, which include the vast majority of the Asia Pacific population, are at least 1% and 7.5% respectively, adjusting for age and stage distribution and predominant cancer types.^9^, ^12^ In terms of the total number of patients receiving radiotherapy, optimal radiotherapy utilisation rate in the Asia Pacific region as of 2020 is 49.1%, translating to 4.66 million out of 9.48 million new cancer diagnoses annually.^13^ A rough extrapolation reveals that approximately 46,000 to 350,000 of them may benefit from PBT, pending more precise modelling studies. Whether they can ultimately receive PBT in their own country or not is another issue, but we posit that these patients should, at the very least, have avenues to access PBT when necessary. We suggest steps moving forward.

First, referral systems among Asia Pacific radiation oncology networks must be established to facilitate coordination of care of patients necessitating PBT. Through organisations like the Federation of Asian Organizations for Radiation Oncology (FARO) and the South East Asia Radiation Oncology Group (SEAROG) that connect radiation oncologists from Asia Pacific countries, collaborative PBT programmes can be formulated to streamline patient referrals within the region and offer logistical support. This provides a pathway by which patients can receive PBT despite unavailability in their country while ensuring that care is delivered timely and efficiently. Additionally, maintaining a strong regional network fosters multi‐institutional research and quality improvement in radiation oncology practice especially in areas which lack expertise in PBT. Individual partnerships with hospitals having PBT facilities represent a step in the right direction, but they must be scaled up and expanded.

Second, in anticipation of the rapid growth in the PBT landscape, individual systems may consider early feasibility studies, policy evaluations, and provisions for overseas treatment without necessarily competing with efforts to improve access to conventional radiotherapy. Engagement between radiation oncologists and public and private insurance companies is necessary in order to draft policies grounded on robust clinical evidence. In Singapore for instance, the Ministry of Health lists approved indications for PBT and their corresponding government insurance claim limits, guaranteeing coverage for eligible patients while setting practice parameters for prescribing physicians.^14^ In Australia where there is no PBT facility, patients can apply to the federal government's Medical Treatment Overseas Programme which provides funding and logistical support for PBT overseas.^15^ Efforts to democratise PBT in Asia Pacific have to take into account its affordability to the general population by minimising out‐of‐pocket expenses as much as possible and avoiding risks of financial catastrophe for vulnerable patients and their families.

Lastly, inter‐regional collaboration between Asia Pacific and western organisations must be strengthened for continued technical and capacity support. As more Asia Pacific countries are investing into proton facilities, coordination among physicians, physicists, therapists and engineers is needed to sustain the collective learning curve on PBT in terms of planning, treatment, workflows and operations. The Particle Therapy Co‐Operative Group, together with the American and European radiation oncology societies, is an excellent platform to share experiences, resources and technical improvements among PBT users worldwide.

Of note, to advocate for improved access to PBT is not to discount the fact that there are much larger issues surrounding cancer care in many Asia Pacific countries. Between protons and conventional x‐ray radiotherapy, the latter remains the standard of care for the most common cancers in the region (and globally), especially in the metastatic setting which ultimately leads to mortality. Investments in cancer screening, early diagnosis and conventional radiotherapy infrastructure, specifically in LMICs, must therefore take precedence in order to make significant improvements in cancer outcomes. Indeed, PBT can benefit some; but better access to conventional x‐rays will benefit most, if not all patients requiring radiotherapy. While we underscore that PBT should not come at the cost of other aspects of cancer care in developing nations, we also recognise that as Asia Pacific economies strengthen, there will be a burgeoning demand for more sophisticated radiotherapy techniques such as PBT. Thus, part of a robust cancer care system should be avenues through which patients who stand to benefit from PBT can access these treatments, without compromising broader cancer control. Improving access to PBT and conventional x‐ray radiotherapy is not mutually exclusive; rather, improving both must occur within the wider context of advancing cancer care in the region.

The importance of cancer care as a critical component of improving overall health and well‐being in Asia Pacific cannot be overemphasised, as it relates to labour productivity and regional economic growth. Paramount in addressing the cancer burden in Asia Pacific is ensuring availability and accessibility of basic cancer care facilities while having the opportunity for patients to have equitable access to modern technologies when necessary. With the exponential growth in the PBT landscape expected in the near future, the gap between the number of patients who can benefit from PBT and those who have access to it must be addressed. This gap is especially large in Asia Pacific, and efforts to bridge it may have far‐reaching implications in cancer care delivery and outcomes in the region. Collaboration and discourse among nations, stakeholders and the global community are crucial to mitigate disparities in access to PBT in Asia Pacific and the rest of the world.

## Funding Information

No funding received for this work.

## Conflict of Interest

The authors declare no conflict of interest.

## Data Availability

Data sharing not applicable to this article as no datasets were generated or analysed during the current study.
